# Immunolocalization of matrix polysaccharides during wood decay by white rot fungus: evidence for specific interaction between hemicellulose and lignin in the wood fibre cell wall of *Dalbergia sissoo* Roxb

**DOI:** 10.3389/fpls.2026.1722528

**Published:** 2026-02-12

**Authors:** Pramod Sivan, Kishore S. Rajput, Karumanchi S. Rao

**Affiliations:** 1 Division of Glycoscience, Department of Chemistry, KTH Royal Institute of Technology, AlbaNova University Centre, Stockholm, Sweden; 2 Department of Botany, Faculty of Science, The Maharaja Sayajirao University of Baroda, Vadodara, India; 3 Department of Biosciences, Sardar Patel University, Vallabh Vidyanagar, India

**Keywords:** cell wall polymers, fungal wood decay, preferential delignification, simultaneous decay, immunolocalization, matrix polysaccharides, lignin

## Abstract

Alterations in the structure and chemistry of cell wall polymers during wood decay by white-rot fungi could be one of the best experimental systems to studying the association between different cell wall polymers and the biology of plant-microbe interactions. We investigated the spatial and temporal changes in the distribution patterns of matrix polysaccharides and lignin in the fibre cell walls of *D. sissoo* wood subjected to preferential delignification and simultaneous decay by two species of white rot fungi. Transmission electron microscopy analysis of fibre walls affected with *L. betulina* showed removal of lignin from the S_1_ layer of the secondary walls (SW), resulting in cell separation. Subsequently, preferential removal of lignin from the S_2_ and S_3_ layers was observed. The structural changes in the SW of fibres inoculated with *D. flavida* directly correlated with the simultaneous degradation of all wall polymers. Immunogold labelling-TEM analysis revealed degradation of xyloglucan in the compound middle lamellae (CML) region, undergoing preferential delignification. Weak labelling for less substituted heteroxylans was evident in S_2_ and S_3_ layers of preferentially delignified fibre walls. Highly substituted heteroxylans showed a higher distribution in the outer layers of SW even at late stages of degradation. The degradation pattern of cell wall polymers suggests a close association between lignin-heteroxylans in the SW as they were removed simultaneously during preferential delignification in the xylem fibres.

## Introduction

Microbial degradation of lignocellulose by white rot fungi has been a major area of research for several decades due to the unique ability of fungi for preferential delignification without affecting the cellulose content. Delignification is an important step for industrial purposes like pulping processes and in the biochemical conversion of lignocellulose into biofuels or commodity chemicals (Blanchette, 1984; Daniel, 2014; Loque et al., 2015). From the ecological perspective, white rot fungi play a pivotal role in the carbon cycle due to their unique enzymatic mechanism to depolymerize lignin and hence increase accessibility to carbohydrates in the cell wall (Fernandez-Fueyo et al., 2012; Hori et al., 2013). The functional diversity of forest fungi is mainly determined by the plant cell wall decomposing machinery (Eastwood et al., 2011). Wood decay by white rot fungus occurs in two different patterns: i) simultaneous decay of all cell wall polymers or ii) preferential delignification of the cell wall. These two different modes of decay are mainly characterized by their specific anatomical features in the wood cell wall structure. Therefore, micro-morphological and ultrastructural studies are the most important tools to assess the preferential delignification or simultaneous decay mechanism of white rot fungus.

During preferential delignification of wood by white rot fungi, the matrix polysaccharides closely associated with lignin are also believed to be degraded by the fungi (Schwarze et al., 2004, 2007). Biochemical studies in degraded wood indicate that cell wall polysaccharides can be degraded simultaneously with lignin. Blanchette (1984) showed that hemicelluloses are removed alongside lignin during wood decay by white rot fungi. Hemicelluloses are believed to play a major role in the organization of cell wall structure by forming tight association with cellulose microfibrils through supramolecular interactions (e.g., hydrogen bonding and non-polar interactions) and with lignin by chemical bonding through lignin carbohydrate complexes (LCC) (Pu et al., 2013; Zhao and Chen, 2014; Ma et al., 2015). Due to the highly heterogeneous chemical makeup of hemicellulose, the chemical interaction of a specific form of hemicellulose with lignin is not fully understood. Xyloglucan (in the primary wall) and glucuronoxylan (in the secondary wall) form the major hemicelluloses in hardwood species. Since secondary wall (SW) represents a major part of biomass, the glucuronoxylan represents the most abundant hemicellulose in the cell wall matrix of hardwoods. Three types of xylans have been proposed in the cell wall of flowering plants: free glucuronoxylan (GX) coated tightly on the surface of cellulose microfibrils, and GX attached to lignin in LCCs through bonds such as benzyl ether, gamma ester, and phenyl glycoside linkages (Ma et al., 2015; Zhang et al., 2017; Martinez-Abad et al., 2018). Hardwood GX exhibits distinct structural motifs in terms of the relative abundance of acetylation and glucuronidation. The decorations make the hydrophilic xylan surface more hydrophobic in case of acetate groups and acidic in case of glucuronic acid groups (Simmons et al., 2016). On a xylan backbone in the ribbon-like 2-fold helical screw conformation, all the decorations that are evenly spaced as in the major domain will face the same side, thus creating a compatible region for hydrogen bonding to the hydrophilic surface of cellulose microfibrils (Busse-Wicher et al., 2014; 2016; Simmons et al., 2016). NMR analysis of GX populations from birch wood (Martínez-Abad et al., 2018) revealed that easily extractable GX interacts with lignin by gamma ester linkages between 4-O-methyl glucuronic acid (mGlcA) and gamma carbon of lignin, while the recalcitrant GX populations correlate with higher amounts of benzyl ether LCCs. These findings indicate a distinct role of the xylan substitutions in the interpolymer interaction during the lignification process, which in turn influences their recalcitrance to processing.

Advancements in high-resolution imaging techniques, such as Raman microscopy and electron microscopy considered to be the reliable tools to study the cell wall polymer changes during microbial degradation in wood tissue (Fleurat-Lessard et al., 2023; Füchtner and Thygesen, 2023; Singh et al., 2024). Immuno-electron microscopy (IEM) with specific antisera raised against cell wall polymers is an effective tool to study their micro-distribution pattern in the cell walls. IEM studies have been extensively used to investigate the spatial and temporal difference in micro-distribution of hemicelluloses and β-(1,4)-galactan in different softwood species (Awano et al., 2002; Altaner et al., 2010) and temperate hardwood trees (Kim and Daniel, 2012; Kim et al., 2012; Sivan et al., 2025). Compression and tension wood have been investigated as well, to understand the spatial variation of cell wall constituents within the cell wall variants of the same cell type (Kim et al., 2010, 2011; Arend, 2008; Mast et al., 2009; Pramod et al., 2014). The immuno-localization of ligninolytic enzymes such as laccase, lignin peroxidase, and manganese peroxidase during advanced stages of wood degradation has been investigated as well by IEM techniques (Blanchette et al., 1989; Daniel et al., 1991; Nicole et al., 1992). However, the organization of cell wall polymers in degraded wood has received little attention so far. We hypothesize that the use of IEM of wood decayed by white rot fungus may help us to understand the possible chemical association between cell wall polysaccharides and lignin. Recent immunocytochemical studies on wood decay in Norway spruce and European ash wood suggested that lignin degradation occurs prior to hemicellulose deconstruction during preferential delignification by *Pycnoporus sanguineus* (Kim and Daniel, 2015a, b). To the best of our knowledge, no detailed information is available on the effect of two modes of white rot decay on the micro-distribution of hemicelluloses, β-(1-4)- galactan, and lignin in the tropical hardwood species.

*Dalbergia sissoo Roxb.*, also known as Indian rosewood, is a fast-growing, deciduous hardwood species with high timber value and is extensively used in furniture, flooring, and plywood. Anatomically, diffuse porous rosewood is comprised of vessels (solitary and radial multiples), fibres (thick-walled with narrow lumen), ray parenchyma, and axial parenchyma cells (Rao et al., 2004). Being a typical hardwood, the xylem cell wall is mainly composed of cellulose, hemicellulose, and lignin (Zhong and Ye, 2015; De Vries et al., 2021). During the primary growth stage, the xylem cell wall is rich in pectins (homogalacturonan and rhamnogalacturonan with β-(1,4)- galactan) and hemicelluloses (mainly xyloglucan) (Cosgrove and Jarvis, 2012; De Vries et al., 2021). The secondary wall of xylem is composed of cellulose, lignin, and hemicellulose (mainly glucuronoxylan and low amounts of glucomannans) (Zhong and Ye, 2014; Sivan et al., 2024). In the present study, we investigated the pattern of lignin degradation and the immunolocalization of heteroxylans (LM10 and LM11), xyloglucan (CCRCM1), and β-(1-4)-galactan (LM5) in the wood fibres of *D. sissoo* subjected to simultaneous decay by *Daedalea flavida Lév* and preferential delignification by *Lenzites betulina* (L.) Fr., using light and transmission electron microscopy (TEM).

## Materials and methods

### Collection and isolation of fungi

Fruiting bodies of white rot fungi, viz., *Daedalea flavida* Lév [synonym *Daedaleopsis flavida* (Lev.) A. Roy and A. Mitra] and *Lenzites betulina* (L.) Fr. were collected from dead wood logs of unknown trees lying on the forest floors at Eturnagaram forest of Andhra Pradesh state, southern India. These samples were immediately packed in sterile polyethylene bags for the isolation and purification of the fungi in the laboratory. A small piece of the fruiting body was dipped in 0.01% mercuric chloride to remove surface contamination and washed several times with distilled water to remove the traces of mercuric chloride. Samples are then transferred aseptically onto 3% malt agar slants. Inoculated tubes were incubated at 28° C for 5–7 days in the incubator. The mycelium collected from the growing edge of those slants was further transferred onto new malt agar slants and incubated under the same controlled conditions to obtain pure cultures. Identification was confirmed using morphological and molecular methods as described earlier (Pramod et al., 2015).

### Plant materials

Wood samples collected freshly from 5 cm thick branches of *Dalbergia sissoo* Roxb., trees growing in the campus of the Department of Biosciences, Sardar Patel University, Gujarat, India. Samples were harvested from branches of three trees having similar age. From each tree, two branches of same thickness were collected to prepare the sample wood blocks. The samples were subjected to light microscopy analysis to check the occurrence of tension wood/gelatinous fibres (G-fibres). The samples without G-fibres were sawn into 20 x 20 × 20 mm cubic blocks and dried in an oven at 50° C for *in vitro* experiments.

### *In vitro* decay test

For the *in vitro* decay test, oven-dried cubic blocks (20 × 20 × 20 mm) were rewetted by soaking in water for 24 hours to obtain the optimum moisture level to facilitate mycelial growth. Prior to wetting, some of the blocks were marked for weighing purposes to know the final weight loss after each incubation period. To exclude the possible pathogens in the samples, the wood blocks soaked in water were autoclaved for 30 min at 120°C. The autoclaved wood blocks were surface sterilized with 70% alcohol and inoculated with 4–5 agar discs (10 mm diameter) removed from the actively growing 2-weeks-old pure cultures of *D. flavida* and *L. betulina* (**Supplementary Figure S1**) and incubated for 30, 60, 90, and 120 days at 27 ± 1 °C and 70% relative humidity in an growth incubator (Nova Instruments Ltd. India, Model No. D803). After each incubation period, these test blocks were removed from the conical flasks and cleaned properly to remove mycelia. The marked blocks were oven dried for weighing to determine final weight loss, while the remaining blocks were fixed in formaldehyde-acetic acid-alcohol (FAA, Berlyn and Miksche, 1976) for light microscopy. For the electron microscopy, samples were trimmed into a 2×5 mm size and fixed in a mixture of 0.1% glutaraldehyde and 4% paraformaldehyde in 50 mM sodium cacodylate buffer for 4 hours at room temperature.

### Light microscopy

Samples trimmed into suitable sizes were dehydrated in a tertiary butyl alcohol series and finally embedded in paraffin with a 58-60°C melting temperature. Transverse, tangential, and radial longitudinal sections of 10 µm and 14- 16 µm thickness were taken with a rotary microtome (Zeus 202A, Singapore) and a sliding microtome (Leica SM2000R, Germany), respectively. The dewaxed sections were subjected to differential staining with safranin and fast green FCF combination (Berlyn and Miksche, 1976), mounted in DPX after dehydration in a xylene-alcohol series. Stained sections were observed and photographed using a Leica DM 2000 microscope attached with a Canon DC 150 Digital Camera (Germany).

### Scanning electron microscopy

Samples were cut into blocks of 2-3mm thickness, dehydrated in a graded series of Acetone, Acetone-Amyl acetate, and dried at room temperature. Dehydrated samples were then coated with gold using a Quaram sputter coating unit (Model SC 7610). The samples were observed and photographed from transverse and tangential planes with LEO 440i SEM at 10 KV.

### Sample preparation for transmission electron microscopy

Fixed samples were washed in buffer, dehydrated in a graded series of ethanol (30-95%), 15 minutes each, pure ethanol × 3, each for 20 minutes. After dehydration, samples were infiltrated and embedded in LR white resin as described elsewhere (Pramod et al., 2014).

### Lignin localization

Transverse ultrathin sections of 70-80nm thickness were prepared from the LR white embedded blocks using an ultra-microtome (Ultracut E, Leica, Germany) with a diamond knife. Sections mounted on nickel grids were stained with 0.1% potassium permanganate (KMnO_4_) in citrate buffer for 45 minutes at room temperature for lignin (Donaldson, 1992).

### Immunogold labelling

Ultrathin sections of 90 nm thickness mounted on nickel grids were used for suspension in buffer ‘A’ (composition: Tris-buffered saline containing 1% bovine serum albumin and 0.1% NaN_3_, pH 8.2) for 30 minutes at room temperature. The grids were incubated with LM5, CCRCM1, LM10, or LM11 antibodies (1:20 dilution in buffer A) for 2 days at 4˚C. The labelling method was the same for xyloglucan, except the grids were incubated with goat antimouse secondary antibody labelled with 5nm colloidal gold particle (BB International, UK). After three washings with buffer A for 15 minutes each, the grids were incubated with goat anti-rat secondary antibody labelled with 10-nm colloidal gold particles (BB International, UK) for 2 hours at room temperature. For the control, some sections were also incubated only with the secondary antibody. Finally, the grids were washed in six changes of buffer ‘A’ for 15 minutes each, followed by washing with distilled water. Ultrathin sections were post-stained with 1% KMnO_4_ for 30 minutes, washed in three changes of distilled water for 10 minutes each. All sections were examined under a transmission electron microscope (TEM, Philips, Morgagni M268) at an accelerating voltage of 70 kV. The gold particle density quantification (per ­ µm^2^ area) after LM10, LM11, and CCRCM1 labelling was based on 20 measurements from five images per triplicated sample. All measurements were taken using ImageJ software (National Institutes of Health, USA).

## Results

### Ultrastructure and distribution pattern of lignin, xyloglucan, β-(1,4)-galactan, and heteroxylans in the untreated cell wall of wood fibre

**Figure 1:**
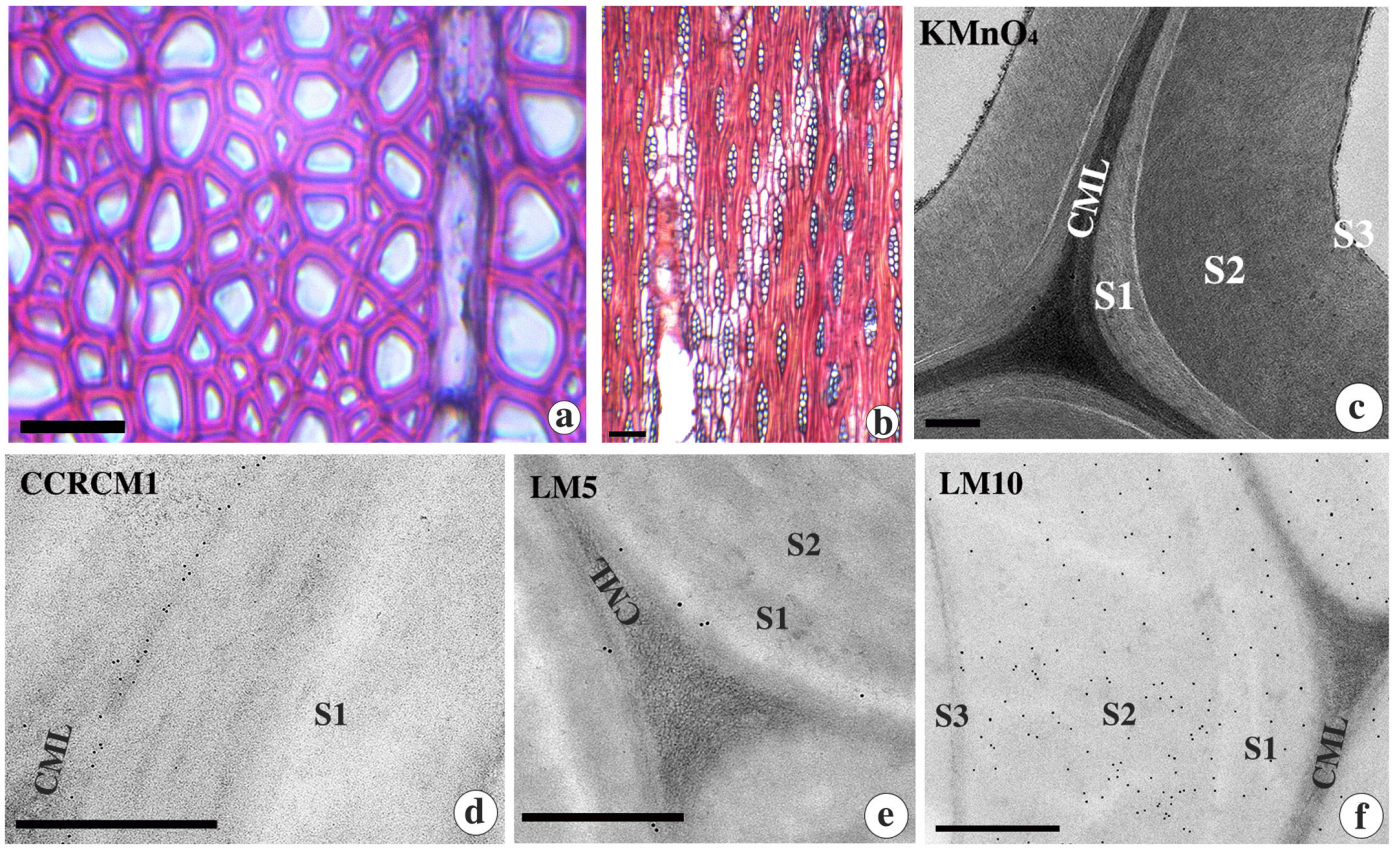
**(a-f)** Light micrographs from the transverse **(a)** and tangential longitudinal **(b)** sections and TEM images **(c-f)** from the transverse sections of control wood tissue of *D*. *sissoo* contrasted with KMnO_4_ **(c)** and immunogold labelling **(d-f)**. Note the secondary wall layers are indicated as S_1_, S_2_ and S_3_. Immunolocalization of xyloglucan with CCRCM1 **(d)**, β-(1-4)-galactan with LM5 **(e)**, and xylan with LM10 **(f)**. CML= compound middle lamellae. Scale bar; a=50 µm, b= 25 µm, c-f = 0.5µm.

Light microscopy of control wood showed the distribution of thick-walled vessels, fibres and relatively thin walled axial and uni-biseriate ray parenchyma cells (**Figures 1a, b**). TEM imaging of KMnO_4_ stained ultrathin sections revealed the fibre cell wall with high lignin distribution in the cell corners and the compound middle lamellae (CML) (**Figure 1c**). The three-layered SW showed differential lignin distribution in each layer. The S_1_ layer had relatively less lignin compared to the S_2_ layer, while the thin S_3_ layer was highly lignified among SW layers (**Figure 1c**). Immunolocalization of xyloglucan (CCRCM1) and β-(1,4)-galactan (LM5) showed that their distribution is limited to the CML region (**Figures 1d, e**). On the contrary, heteroxylans (LM10) were found exclusively in the secondary cell wall (**Figure 1f**).

### Simultaneous degradation of the cell wall by D. flavida

#### Pattern of mycelial entry and cell wall degradation

**Figure 2:**
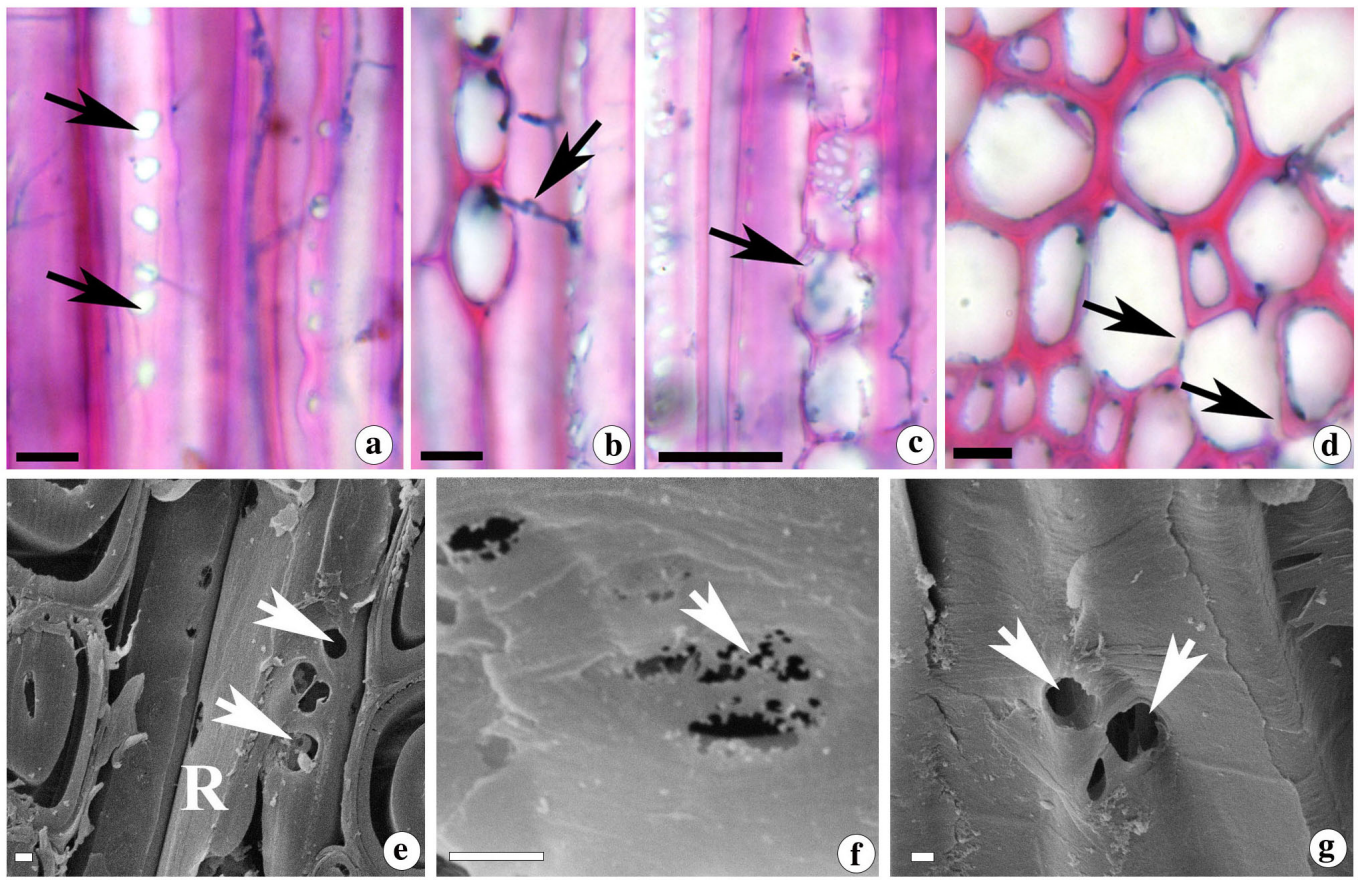
**(a-g)** Light **(a-d)** and scanning electron micrographs **(e-g)** from the tangential longitudinal **(a-c, f-g)** and transverse sections **(d-e)** of *D*. *sissoo* wood inoculated with *D.flavida.* **(a, e)** Boreholes formed in the fibre wall (arrows) and pit (arrowheads) **(b)** fungal mycelia (arrow) migrating between fibre to ray and **(c)** between ray cells. **(d, f)** thinning of fibre cell wall during simultaneous decay and **(g)** formation of large void areas (arrows). Treatment duration of samples; **(a-c, e)** are from 60 days and d,f and G are from 90 days. R = ray cell. Scale bar = 10 µm **(a-d)**, 1 µm **(e-g)**.

Bright field and scanning electron microscopy revealed the sequential stages of entry of fungal mycelia between cells and subsequent cell wall degradation. The wood inoculated with *D. flavida* showed the formation of large bore holes which facilitates the migration of mycelia between fibres (**Figure 2a**), from fibres to ray (**Figure 2b**) and between ray cells (**Figure 2c**). The simultaneous degradation of cell wall polymers led to thinning of fibre SW (**Figure 2d**). SEM also confirmed the borehole formation throughout the fibre length through simultaneous cell wall degradation, leading to thinning and formation of void regions in the cell wall (**Figures 2e-g**).

#### Ultrastructural changes in the degraded cell wall

**Figure 3:**
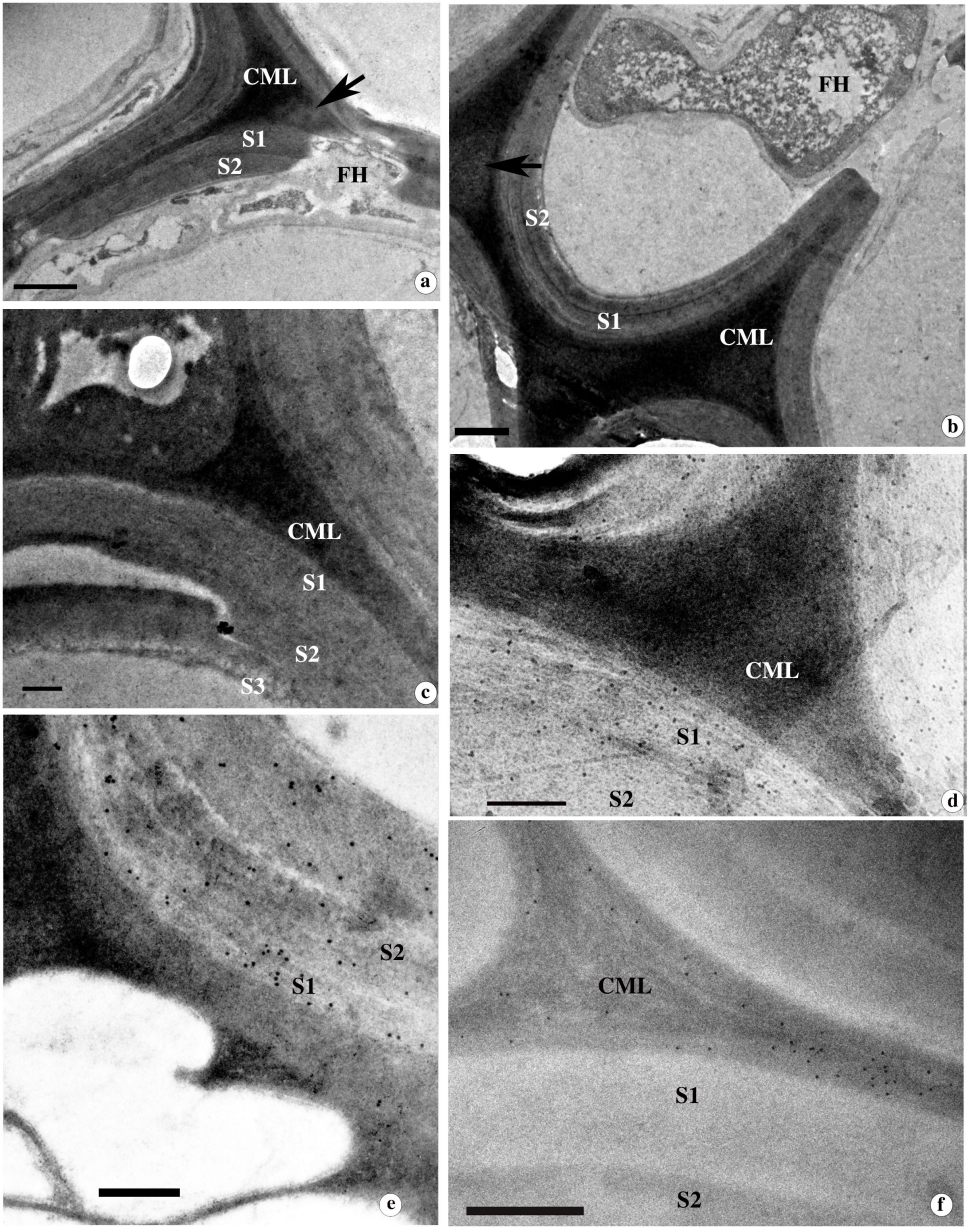
**(a-f)** TEM images from the transverse section of *D*. *sissoo* wood inoculated with *D.flavida* for 90 days contrasted with KMnO_4_ ***(*a,b*)*** and immunolocalization of β-(1,4)-galactan with LM5 **(c)**, xylan with LM10(d) and LM11 **(e)**, and xyloglucan with CCRCM1 **(f)**. ls ACG Xs = low substituted xylan; hs ACG Xs = highly substituted xylan. **(a)** Localized degradation of SW and CML of the fibre cell wall. Note the fungal hyphae (FH) attached to the inner SW facing cell lumen surrounded by a hyphal sheath. **(b)** Spreading of fungal hyphae through simultaneous degradation of the cell wall between fibres. The arrow indicates the electron translucent regions in the cell corner formed during the delignification process. FH = Fungal hyphae hyphal sheath. **(c)** CML and SW regions of degraded fibre cell wall showing absence of labelling for β-(1-4)-galactan. **(d)** The SW of fibre wall showing strong labelling for ls ACG Xs. Note the absence of gold labelling in the CML region. **(e)** The S_1_ layer and outer regions of the S_2_ layer of the fibre cell wall showing intense labelling with LM11 antibody. **(f)** Relatively more xyloglucan labelling in the CML of fibre wall undergoing simultaneous decay by *D.flavida.* Scale bar= 1µm; SW=Secondary wall.

The ultrastructural changes during simultaneous degradation and the associated delignification process were observed using TEM analysis of transverse sections stained with KMnO_4_. Thinning of the inner layer of the SW in contact with the fungal hyphae was noticed from the early stages of degradation. Thinning occurred either along the inner circumference of the cell wall or locally (**Figures 3a, b**). Relatively less electron-dense hyphal sheath or slime layer was apparent around the hyphae observed in the lumina of cells (**Figure 3b**). When simultaneous degradation of the SW progressed, delignification was observed in the lignin-rich CML, resulting in the formation of electron translucent void areas in the cell corner and CML of locally degraded cell wall areas (**Supplementary Figures S2a, c**). Due to the localized simultaneous decay, the SW of fibres often appeared wavy (**Supplementary Figure S2c**). At this stage, the SW lost the integrity of its interlinked cell wall polymers, leading to the separation of cell wall layers. The loss of integrity of cell wall polymers was evident from the splitting of SW within the S_2_ layer or the boundary of the S_1_ layer during the progression of the delignification process (**Supplementary Figure S2b**).

#### Immunolocalization of β-(1-4)-galactan, xyloglucan, and heteroxylans in the simultaneously degraded cell walls

**Figure 4:**
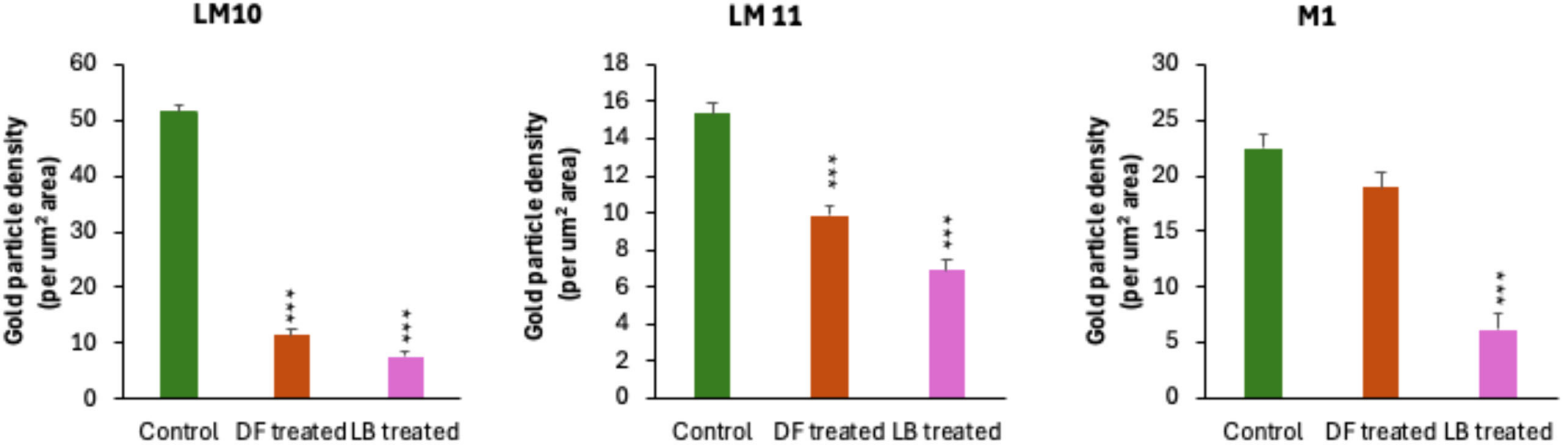
Density of gold particles (per mm^2^ area) in the control, *D. sissoo* wood inoculated by *L. betulina* (LB) and *D. flavida* (DF) for 90 days using LM10, LM11, and CCRCM1 (M1). ***P ≤ 0.001 for comparisons with control by Dunnett´s test.

Localization of β-(1,4)-galactan with LM5 antibody did not show any significant labelling in the cell wall undergoing degradation process (**Figure 3c**). Stronger labelling of less substituted heteroxylans (ls ACG Xs) with LM10 antibody was mainly confined to the SW regions (**Figure 3d**). The CML did not show any xylan labelling with LM10 (**Supplementary Figures S3a, b**). Highly substituted heteroxylans (hs ACG Xs) labelled with LM11 antibody showed strong labelling even in the remnants of SW during advanced stages of decay (**Supplementary Figures S2c, d**). In the fibre cell wall, the maximum gold labelling with LM11 antibody was noticed in the corner region of the SW, suggesting a higher concentration of hs ACG Xs in this area of SW (**Figure 3e**, **Supplementary Figure S3d**). Despite of this spatial distribution variation pattern, effect on overall xylan distribution was evident from the reduction in the density of gold particles per µm^2^ area with LM10 and LM11 labelling (**Figure 4**). CCRCM1 labelling showed the presence of gold particles in the CML region of fibre cell walls (**Figure 3f**), suggesting the retention of xyloglucan in the CML region during simultaneous decay in the secondary wall.

### Preferential delignification of the cell wall by L. betulina

#### Pattern of mycelial entry and cell wall degradation

**Figure 5:**
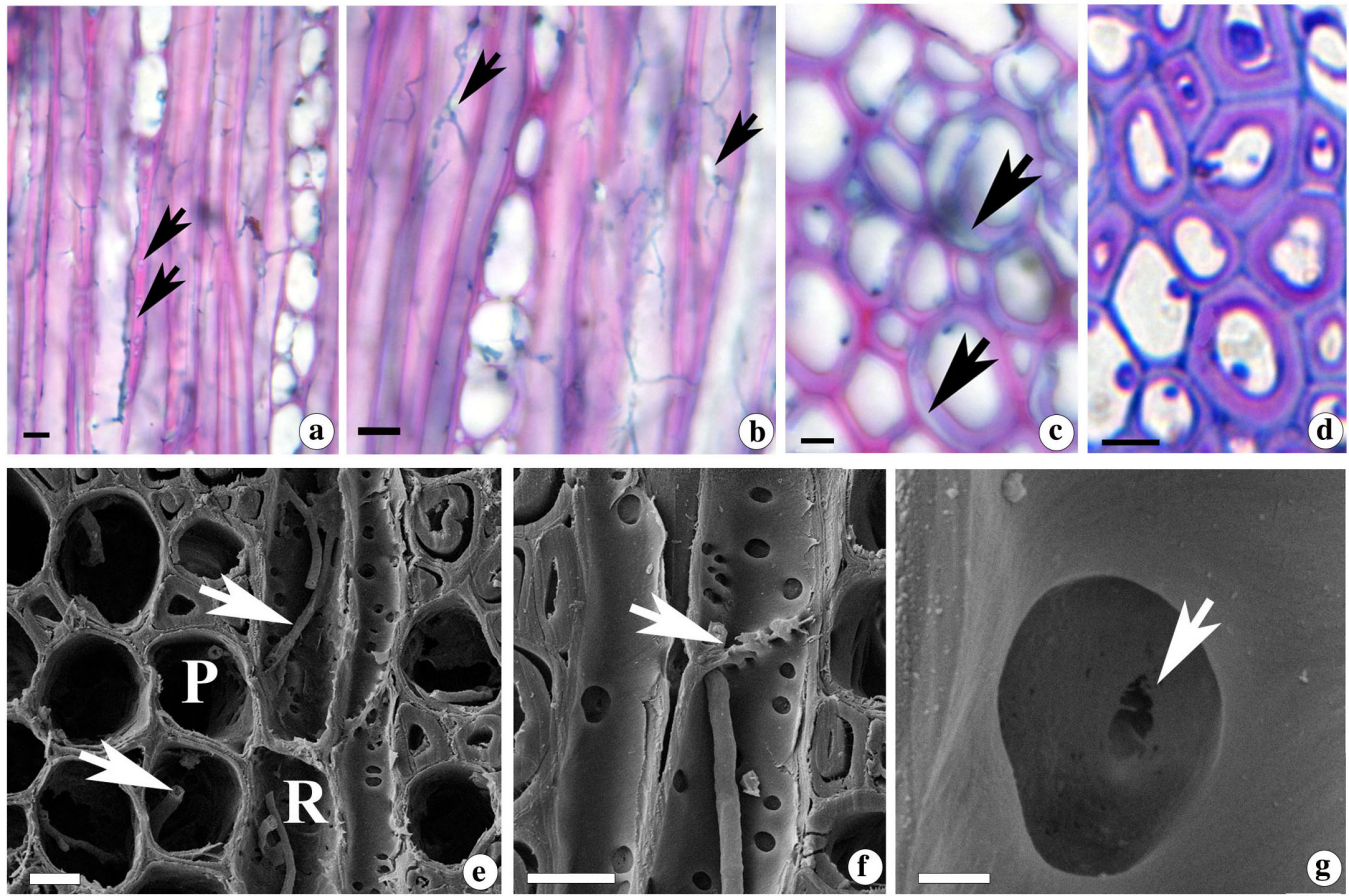
**(a-g)** Light **(a-d)** and scanning electron micrographs **(e-g)** from the tangential longitudinal **(a, b, g)** and transverse sections **(c-f)** of *D. sissoo* wood inoculated with *L. betulina.* **(a, b, e, f)** Migration of fungal mycelia through the pits (arrows); P = Parenchyma, R = Ray cell. **(c)** Section taken by a sliding microtome showing the delignified secondary wall separated from the lignified part of the cell wall (arrows) **(d)** Section from the resin embedded material showing delignified secondary wall (purple colour) remains intact with lignified cell wall (blue colour) **(g)** Pit membrane showing breaks (arrow) during fungal migration into the cell. Treatment duration of samples; **(a-c)** are from 60 days and **(d-g)** are from 90 days. Scale bar = 10 µm **(a-d)**, 10 µm **(e, f)**, g (1 µm). ls ACG Xs = low substituted xylan; hs ACG Xs= highly substituted xylan.

The migration of mycelia occurred through the pit regions of ray cells and fibres (**Figures 5a, b**). Delignification of the outer layers of the SW of fibres was evident from weak staining reaction with safranine (**Figure 5c**). On the other hand, the red staining of the inner SW layer with safranine suggests the presence of lignin. The weak delignified secondary cell wall often detached during sectioning by sliding microtomy. However, the toluidine blue staining of a semi-thin section taken using an ultramicrotome from the resin-embedded material revealed the intact delignified SW stained purple and remained attached to compound middle lamellae (**Figure 5d**). Partial delignification has been reported to cause separation of the S_2_ and S_3_ layers from the S_1_ layer (Brändström et al., 2003). SEM imaging revealed the migration of fungal hyphae through the pit by breaking the pit membrane while the cell wall remained intact (**Figures 5e-g**).

#### Ultrastructural changes in the cell walls of wood

**Figure 6:**
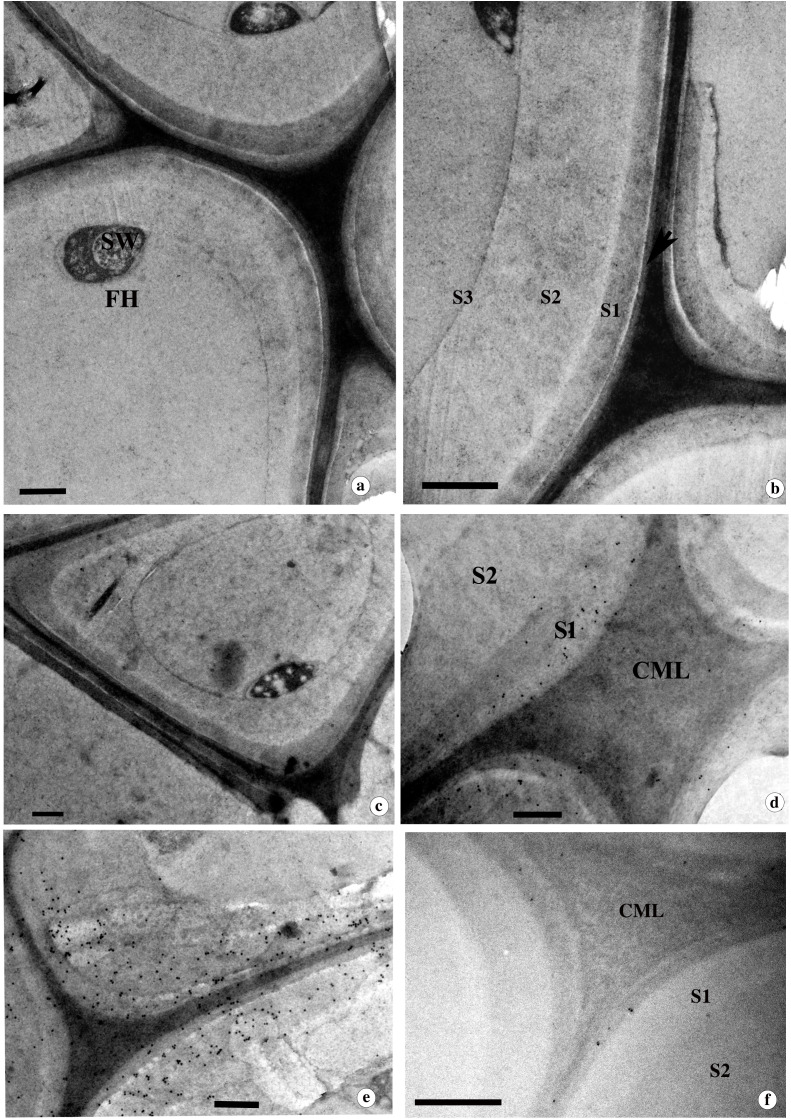
TEM images from the transverse section of *D*. *sissoo* wood inoculated by *L. betulina* for 90 days contrasted with KMnO_4_ **(a, b)** and immunolocalization of β-(1-4)-galactan with LM5 **(c)** and xylan with LM10(d), LM11 **(e)** and xyloglucan with CCRCM1 **(f)**. **(a)** Preferential delignification of the secondary wall region of fibre showing loss of staining contrast with KMnO_4_. Note the fungal hyphae (FH) attached to the inner secondary wall. **(b)** Fibre cell wall showing progression of delignification in the S1 layer and CML region. Note the electron-dense cell corner middle lamellae region. **(c)** Fibre cell wall undergoing preferential delignification at CML and SW showing weak β-(1-4)-galactan labelling. **(d)** The corner region of the secondary wall (SW) of the fibre showing xylan labelling with LM10 antibody. Note the weak labelling from the preferentially delignified inner SW regions. **(e)** The preferentially delignified SW showing strong labelling of hs ACG Xs in S1 and the outer region of the S_2_ wall layer. Note the strong labelling from the CML region undergoing delignification. **(f)** Weak xyloglucan labelling in the CML of fibre wall undergoing preferential delignification by *L. betulina*. Scale bar = 1µm.

The light microscopy indications on preferential delignification of fibre cell wall were further confirmed by the weak contrasting of secondary wall with KMnO_4_ (**Figure 6a**). Delignification began within a short period of fungal inoculation. At the end of 30 days of incubation, delignification was noticed in the cell corners and the CML region. Subsequently, patches of electron-translucent areas appeared following preferential delignification (**Figure 6b**).

#### Immunolocalization of β-(1,4)-galactan, heteroxylans, and xyloglucan in the preferentially delignified cell walls

The cell wall of fibres undergoing preferential delignification did not show strong β-(1,4)-galactan labelling with LM5 antibody (**Figure 6c**). LM10 antibody labelling showed that a higher distribution of ls ACG Xs is mainly confined to the S_1_ layer of the SW (**Figure 6d**), while CML and the rest of the SW regions showed weak labelling. The loosely organized, delignified regions of secondary cell wall showed the absence of labelling, suggesting removal of ls ACG Xs along with lignin during delignification (**Supplementary Figures S4a, b**). The delignified, electron translucent regions of CML did not show any xylan labelling with LM10 antibody during advanced stages of decay (**Supplementary Figure S4**). Localization of highly substituted heteroxylans (hs ACG Xs) with LM 11 antibody showed a strong labelling from the outer part of the S_2_ and S_1_ layers of the secondary cell wall (**Figure 6e**). Strong labelling was also observed in the CML region (**Figure 6e**). A gradual decrease in the density of gold particles was noticed (**Supplementary Figure S5**), corresponding to the decrease in contrasting with KMnO_4_ staining in the SW of fibres undergoing delignification. Weak labelling of hs ACG Xs was also observed in CML regions undergoing active delignification (**Supplementary Figure S5d**). CCRCM1 labelling revealed weak labelling in the CML region (**Figure 6f**), indicating effective removal of xyloglucan during preferential delignification by *L. betulina*. The sequential stages of delignification and associated changes in the distribution of heteroxylans localized by LM10 and LM11 labelling are provided in **Supplementary Figures S3**, **S4**, respectively. The decrease in density of gold particles for LM10, LM11, and M1 labelling was relatively more intense in the wood treated with *L. betulina* (**Figure 4**).

## Discussion

### Anatomical changes during simultaneous degradation and preferential delignification of wood

Light and TEM analysis of fibre cell wall degraded by *D. flavida* revealed the typical anatomical and ultrastructural features associated with simultaneous decay, especially the thinning of secondary cell wall regions in contact with the fungal hyphae and localized removal of middle lamellae (Anagnost, 1998; Bhatt et al., 2016). The advancement of thinning took place either locally or along the inner circumference of the SW. Blanchette (1991) reported that the thinning and general erosion of the cell wall by non-preferential white rot fungi involves an array of lignin and cellulose-degrading extracellular enzymes. Hyphal sheaths of *D. flavida* were present within the lumina of wood cells. The sheath formed around the wood decaying fungal hyphae may serve as a medium for housing along the surface of the cell wall, transporting lignocellulose depolymerizing agents, as a source of support and nutrition (Blanchette and Reid, 1986; Green et al.,1992; Ruel and Joseleau, 1991; Blanchette et al., 1997). *D. flavida* caused simultaneous decay in *Dalbergia* wood; the strong ligninolytic ability of this fungus was evident from the appearance of an electron-translucent void region due to degradation of lignin in the cell corner and CML regions of fibres. The type of lignin found within different regions of the cell wall may influence the degradation pattern. The cell corners of hardwood are generally characterized by high guaiacyl lignin content, while other morphological regions have a greater fraction of syringyl lignin (Blanchette and Reid, 1986). White rot fungi that attack various hardwoods appear to preferentially degrade the syringyl lignin component (Highley, 1982). The occurrence of delignification of cell corner regions of fibres by *D. flavida* suggests the efficiency of this fungus to degrade guaiacyl lignin units.

The TEM analysis revealed the intact secondary cell wall with weak contrast for the KMnO_4_ method in the wood treated by *L. betulina*, indicating the effective preferential delignification without damaging the carbohydrate skeleton. The interfibrillar texture of cell corner CML is masked by monolignols and embedded within the lignin matrix during cell wall maturation (Kim et al., 2015). The present study showed the appearance of electron translucent regions in the CML region, which also indicates preferential delignification. The lack of contrast with KMnO_4_ staining in degraded wood is a clear indication of the attack of fungal enzymes from the cell lumen inward towards the middle lamellae region (Blanchette and Reid, 1986). Unlike *D. flavida*, degradation of the cell corner region of fibres and ray cells was not observed in the wood decayed by *L. betulina*. These observations suggest that *L. betulina* is similar to other white rot fungi, causing preferential delignification in hardwood species (Faix et al., 1985; Highley, 1982; Kirk, 1987). These white rot fungi, causing preferential delignification, may have an enzymatic mechanism that is less efficient in degrading guaiacyl lignin present at the cell corner region of fibres and ray cells. However, axial parenchyma cells showed delignification of cell corner regions, plausibly due to variation in lignin monomeric composition in the cell corner region between different cell types.

### Changes in the cell wall polymers during simultaneous degradation and preferential delignification

Pectins and xyloglucans (hemicellulose) form the major matrix polysaccharides in the primary cell wall (Park and Cosgrove, 2015; De Vries et al., 2021; Sivan et al., 2024). The hypothesis on pectin-lignin interaction in the cell wall has been proposed based on the distribution pattern of high pectin in the middle lamellae of differentiating fibres where lignification is initiated and more intense (Wi et al., 2005). In *Arabidopsis*, the developmental disappearance of the LM5 epitope reflects degradation of pectic β-(1, 4)-galactan or the modification/masking of the epitope in the cell wall (McCartney et al., 2003). In the present study, we could not observe any gradual change, and labelling was weak for β-(1,4)-galactan in the CML region during early stages and advancement of preferential delignification. This suggests that β-(1,4)-galactan may be present in much less quantity in the CML region of mature fibres of *D. sissoo*, or that they might have been degraded along with lignin in the early stages of cell wall degradation. Xyloglucans (XyG) contribute to the biomechanical hotspot model for primary cell walls by mediating contact junctions between adjacent cellulose microfibrils (Cosgrov, 2022). Immunolocalization of xyloglucan with CCRCM1 revealed relatively strong labelling for XyG in the CML region of fibre walls undergoing simultaneous decay by *D.flavida*. On the other hand, a rapid degradation of XyG was evident in the CML region of fibre walls undergoing preferential delignification by *L. betulina*. The degradation of XyG during preferential delignification of CML suggests that XyG might be in close proximity to lignin in the primary wall of *D. sissoo* wood fibre.

Heteroxylans are a major hemicellulose component in the secondary cell wall of hardwood fibres (Donaldson, 2009). During preferential delignification in birch wood by *P. tremellosus*, depletion of hemicelluloses along with lignin was apparent from the lumen towards the middle lamellae, suggesting that lignin and hemicelluloses are associated in the matrix that surrounds cellulose fibrils (Blanchette and Reid, 1986). The present study showed the degradation pattern of both low-substituted heteroxylans (ls ACG Xs) and high-substituted heteroxylans (hs ACG Xs) in the fibre cell wall during the decay process. The immunocytochemical studies in both softwood (Norway spruce wood) and hardwood (European ash wood) species subjected to preferential delignification mode of white rot decay by *P. sanguineus*, demonstrated two different types of degradation patterns, the preferential degradation of lignin without changes in cellulose and hemicellulose observed in softwood tracheid while in hardwood, the degradation of lignin proceeds hemicelluloses (Kim and Daniel, 2015a, b). FTIR analysis of beech and scot pine wood subjected to preferential delignification by *P. chrysosporium* showed a decrease in xylan content along with lignin, whereas simultaneous decay by *C. versicolor* caused a lignin-carbohydrate reduction at a similar rate (Pandey and Pitman, 2003). Immunogold labelling analysis of poplar wood fibres subjected to delignification by hydrothermal pretreatment reported to remove xylan covalently bound to lignin and those tightly bound to cellulose microfibrillar surface of fibre cell walls (Ma et al., 2015). Our results agree with previous reports on the existence of tight interaction between GX and lignin in the secondary cell wall matrix of hardwood fibres. Since hemicelluloses can act as a physical barrier around cellulose microfibrils, limiting cellulase accessibility during enzymatic hydrolysis (Penttila et al., 2013), *L. belutina* might exhibit strong xylan degrading activity during the preferential delignification process. During simultaneous degradation by *D. flavida*, ls ACG Xs disappeared first, especially in the lignin-rich CML region, whereas hs ACG Xs showed strong labelling even in the remnants of the degraded cell wall. On the other hand, during the preferential delignification, the distribution of ls ACG Xs was higher in the S_1_ layer, while weak labelling was observed in CML and the remaining SW regions. However, hs ACG Xs showed strong labelling in cell corner regions during initial stages of degradation, while their distribution decreased gradually with decay advancement. The hardwood xylan with a closer mGlcA substitution pattern is proposed to tightly interact with lignin (Martinez-Abad et al., 2018; Sivan et al., 2024). Our results indicate that hs ACG Xs with mGlcA substitution pattern might gradually disappear along with lignin during preferential delignification in the CML region.

The pattern of spatial and temporal changes in the matrix polysaccharides during simultaneous degradation and preferential delignification indicates the difference in microbial degradation mechanism and interaction pattern of hemicellulose-lignin in the cell wall of *D. sissoo* wood. Lignin carbohydrate complex in the wood biomass is proposed to be made of three major ester linkages; (1) phenyl glycosides (PG) between hydroxyl groups in the lignin and anomeric carbon in polysaccharides, (2) benzyl ether (BE) between lignin hydroxyl groups and carbons (C2, C3 or C6) in the sugar units of polysaccharides, (3) γ-esters (GE) formed between carboxylic acid group in the mGlcA in the xylan backbone and γ-OH groups in the lignin (Derba-Maceluch et al., 2025; Terret and Dupree, 2019). Cell wall degradation during fungal wood decay has been attributed to abiotic chemistry driven by plant cell wall degrading enzymes (PCDEs) (Riley et al., 2014; Röllig et al., 2025). During brown rot mode of wood decay by *F. pinicola*, the initial phase of cell wall polysaccharide degradation occurs predominantly by Fenton chemistry in the presence of oxygen (O2), while limitation of O2 in the advanced stage is driven by PCDEs such as lytic polysaccharide monooxygenases (LPMOs), class II peroxidases, and hemicellulases (Röllig et al., 2025). The release of mGlcA from birch wood LCC by application of fungal glucuronoyl esterases (GSEs) suggested that about 30-40% of mGlcA of glucuronoxylan could be GE-linked to the lignin (Raji et al., 2021). The recent study on the reduction of GE bonds by secondary wall-specific expression of PcGCE revealed a reduction in xylan content and xylan-lignin cross linking in transgenic aspen trees (Derba-Maceluch et al., 2025). The reduced labelling of xylan (LM10 and LM11) during fungal delignification in *Dalbergia* wood fibres also suggests the possible impact on xylan-lignin linkages, such as GE by fungal PCDEs.

## Conclusion

*In vitro* wood decay experiment in *D. sissoo* using two white rot fungi, *D. flavida* and *L. betulina* demonstrated the simultaneous decay and preferential delignification, respectively. Weak labelling of LM5, LM10, and LM11 antibodies in wood degraded by *D. flavida* suggests the simultaneous removal of cell wall polymers. On the other hand, the gradual change in the labelling pattern of both less and highly substituted heteroxylans indicates that *L. betulina* caused preferential delignification, which is also supported by KMnO_4_ staining contrast in the same cell wall regions. Degradation of xyloglucan from the CML region during preferential delignification suggests that XyG might be in close proximity to lignin in the primary wall. Our results indicate the possibility of a close association between the lignin-xyloglucan in the primary wall and lignin-heteroxylans in the secondary walls as they are removed simultaneously during preferential delignification. Detailed biochemical studies using advanced chromatography-mass spectrometry methods are necessary to confirm the occurrence of such molecular interaction between matrix polysaccharide and lignin.

## Data Availability

The original contributions presented in the study are included in the article/**Supplementary Material**. Further inquiries can be directed to the corresponding author.
