# Supplementary material for: Immunolocalization of matrix polysaccharides during wood decay by white rot fungus: evidence for specific interaction between hemicellulose and lignin in the wood fibre cell wall of Dalbergia sissoo Roxb
Source: Front Plant Sci. 2026 Feb 12;17:1722528. doi: 10.3389/fpls.2026.1722528 (PMC12935940; doi:10.3389/fpls.2026.1722528)
Supplement: Supplementary file 1 [file DataSheet1.docx]

Supplementary Material

## Supplementary Figures


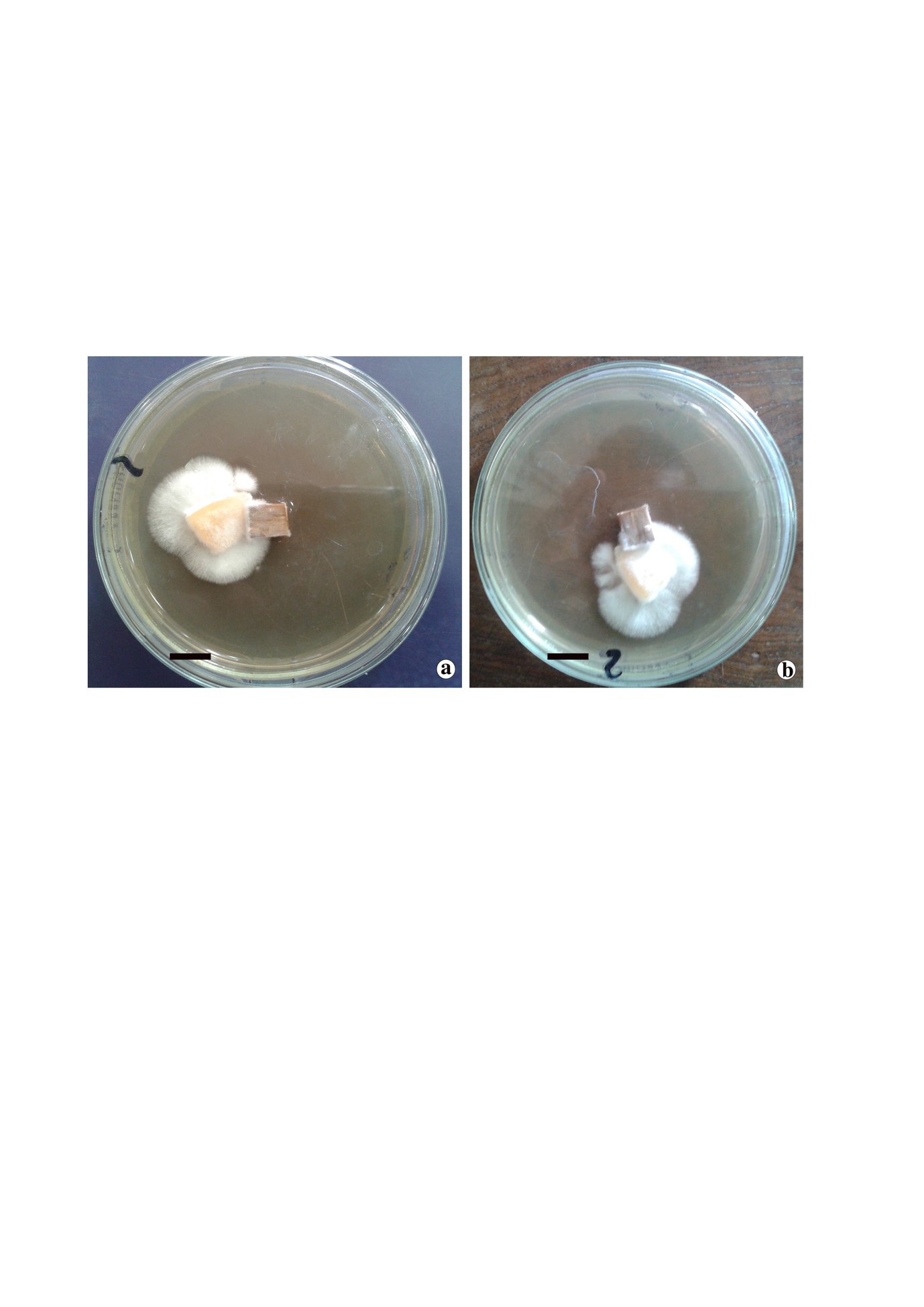


Figure S1: Macroscopic image of *in vitro* experiment showing wood blocks incubated with *D. flavida* (a) and *L. betulina* for 90 days. Scale bar=2 cm.


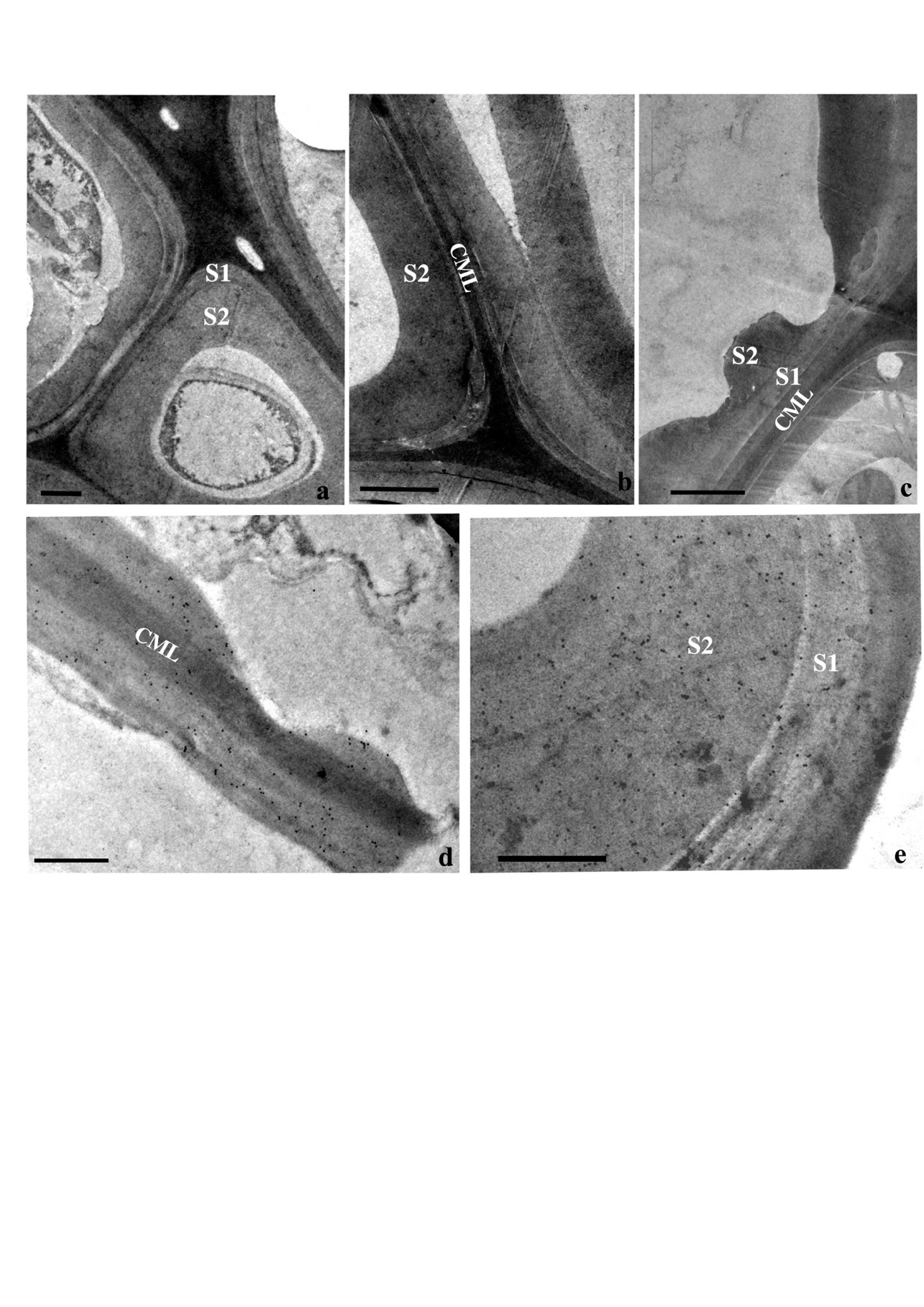


**Figure S2:** TEM images from transverse section of *D. sissoo* wood inoculated with *D.flavida*  for 90 days contrasted with KMnO_4_ (a-c) and immunolocalization of xylan with LM11(d,e). (a) Delignification of CML and secondary wall of fibre cell wall (arrows). Note the electron translucent void regions within the cell corner and CML regions undergoing simultaneous degradation. (b) Splitting of cell wall within S2 layer of SW in fibre. Note the loss of contrast to CML due to progression of delignification process. (c) The wavy appearance of SW following localized degradation of fibre cell wall. (d) The delignified CML region and partially degraded SW showing strong labelling for hs ACG Xs. (e) Corner region of SW of fibre cell wall showing high density of gold labelling for hs ACG Xs. Scale bar= 1µm; SW= Secondary wall.


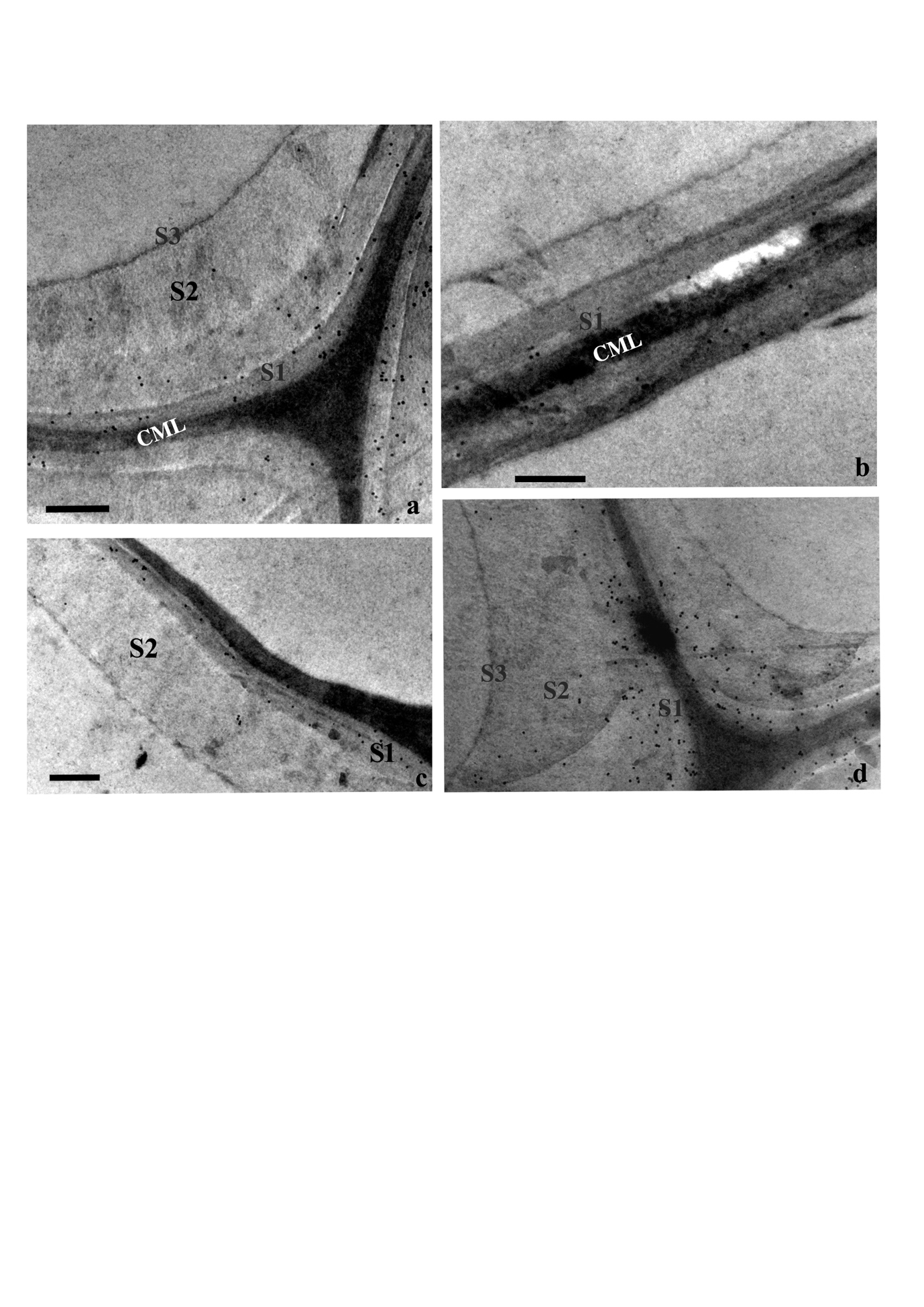


**Figure S3:** Immunolocalization of xylan with LM10 (a) and LM11(b-d) in the cell wall of degraded wood of *Dalbergia* *sissoo* inoculated with *D.flavida* for 90 days. (a) The S1 layer and outer part of S2 layer in fibre SW showing strong xylan labelling. Note the weak labelling from the selectively delignified CML region. (b) Fibre cell wall showing weak labelling with LM10 antibody in the CML region during advanced stage of selective delignification. Note the presence of gold particles in the S1 region. (c) The fibre SW showing weak distribution of hs ACG Xs during advanced stages of decay. Note the labelling is limited to S1 wall layer only. (d) Corner region of SW showing decrease in xylan labelling during late stage of delignification. Note the CML undergoing delignification (arrow). Scale bar= 1µm; SW=Secondary wall.


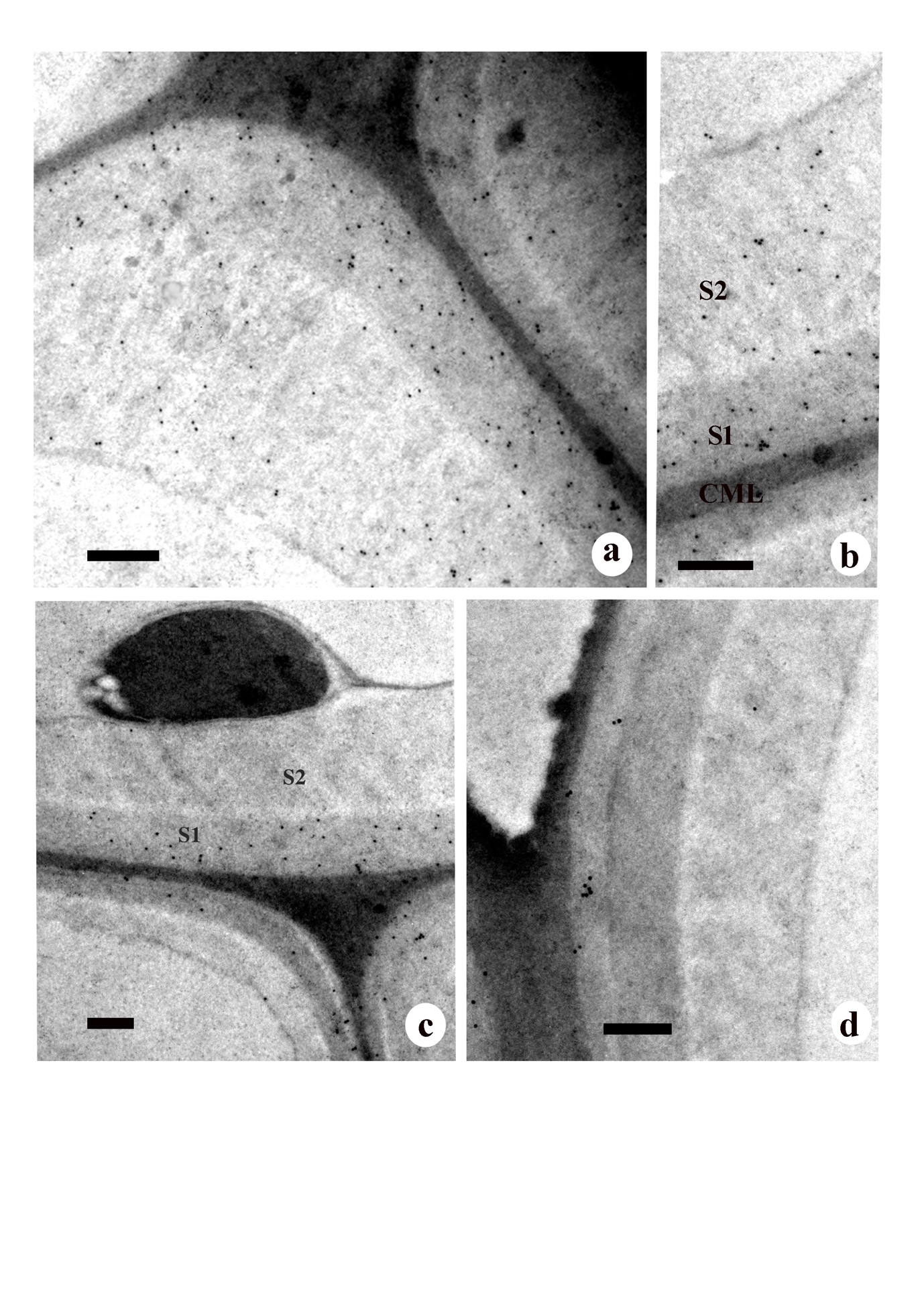


Figure S4 (a-d): Sequential stages of delignification and associated changes in the distribution pattern of low substituted xylans localized by immunolabelling with LM10. Scale bar= 0.5 µm


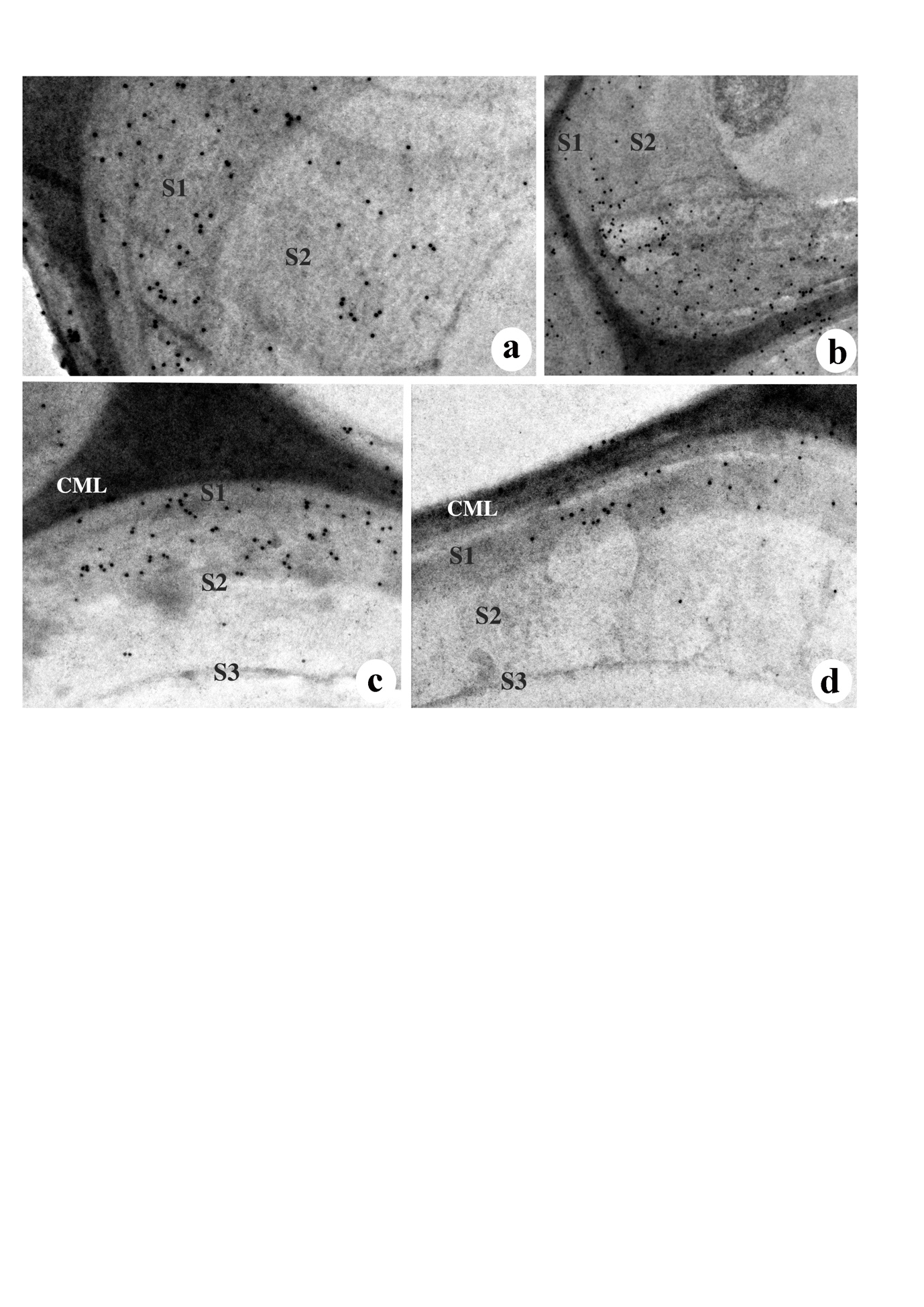


**Figure S5 (a-d):** Sequential stages of delignification and associated changes in the distribution pattern of highly substituted xylans localized by immunolabelling with LM11. Scale bar= 0.5 µm
